# Hepatocellular Carcinoma Emergence in Diabetic Mice with Non-Alcoholic Steatohepatitis Depends on Diet and Is Delayed in Liver Exhibiting an Active Immune Response

**DOI:** 10.3390/cancers12061491

**Published:** 2020-06-08

**Authors:** Mélanie Simoes Eugénio, Muhammad Farooq, Sarah Dion, Christelle Devisme, Céline Raguenes-Nicol, Claire Piquet-Pellorce, Michel Samson, Marie-Thérèse Dimanche-Boitrel, Jacques Le Seyec

**Affiliations:** 1 University Rennes 1, Inserm, EHESP, Irset (Institut de Recherche en Santé, Environnement et Travail)-UMR_S 1085, F-35000 Rennes, France; se_melanie@outlook.fr (M.S.E.); muhammad.farooq@uvas.edu.pk (M.F.); sarah.dion@univ-rennes1.fr (S.D.); christelle.devisme@univ-rennes1.fr (C.D.); celine.raguenes-nicol@univ-rennes1.fr (C.R.-N.); claire.piquet-pellorce@univ-rennes1.fr (C.P.-P.); michel.samson@univ-rennes1.fr (M.S.); 2 Department of Clinical Sciences, College of Veterinary and Animal Sciences, Jhang 35200, Pakistan

**Keywords:** nutrition, NASH, HCC, animal study

## Abstract

The increase of the sedentary lifestyle and high-calorie diet have modified the etiological landscape of hepatocellular carcinoma (HCC), with a recrudescence of non-alcoholic fatty liver disease (NAFLD), especially in Western countries. The purpose of our study was to evaluate the impact of high-fat diet feeding on non-alcoholic steatohepatitis (NASH) establishment and HCC development. Streptozotocin-induced diabetic male mice were fed with high-fat-high-cholesterol diet (HFHCD) or high-fat-high-sugar diet (HFHSD) from 1 to 16 weeks. Even if liver tumors appear regardless of the high-fat diet, two distinct physiopathological patterns were evidenced, with much more severe NASH hallmarks (liver injury, inflammation and fibrosis) in diabetic mice fed with HFHCD. The mild hepatic injury, weak inflammation and fibrosis observed in HFHSD were interestingly associated with earlier emergence of more numerous liver tumors. When activated helper and cytotoxic T cells, detected by flow cytometry, infiltrated the liver of HFHCD-fed diabetic mice, a delay in the appearance of tumor nodules and a limitation of their numbers were observed, suggesting that the immune activities partly controlled tumor emergence. These data highlighted two different mouse models of HCC progression in diabetic mice depending on diet, which could be useful to evaluate new therapeutic approaches for HCC by targeting the immune response.

## 1. Introduction

According to the World Health Organization, the liver is the seventh organ most affected by cancer in the world, but is the fourth leading cause of cancer-related deaths (Globocan2018). Between 85% and 90% of primary liver cancers derived from hepatocytes [1]. Many risk factors for the development of this hepatocellular carcinoma (HCC) are relatively well known. The most important ones include chronic viral hepatitis, linked to Hepatitis B or C virus (HBV or HCV) infections, environmental toxins as aflatoxins, abuse of alcohol consumption, metabolic diseases such as obesity or diabetes mellitus, genetic disorders (Wilson’s disease and hemochromatosis for instance) and more recently non-alcoholic fatty liver disease (NAFLD) [2].

In the past few years, many countries are facing a significant increase in the incidence of obesity and diabetes mellitus [3,4]. The onset of these metabolic diseases is linked to a reduction in physical activity associated with an increase in dietary energy intake [5,6]. These two human diseases are frequently associated with NAFLD [7], which is subdivided in two categories in adults according to their severity. At the first stage, the nonalcoholic fatty liver (NAFL) only shows steatosis. Then, the liver disease progresses to the nonalcoholic steatohepatitis (NASH) phase, characterized by steatosis, liver inflammation and hepatocyte ballooning, which could lead to fibrosis accumulation in the liver parenchyma, ultimately evolving to cirrhosis and HCC [8]. Unlike other etiologies, HCC in patients with NAFLD occur on non-cirrhotic livers in 25–45% of cases. Diet affects not only the occurrence of this disease but also its evolution. Unhealthy eating habits usually involve overconsumption of carbohydrates, red meats and saturated fatty acids, which is generally associated with a decrease in fiber and omega-3 intake. This unbalanced food intake could lead to metabolic disease onset and promotes NAFLD progression [9,10,11].

The liver plays an important role in immunological surveillance, especially for gut-derived products containing many exogenous elements, mainly harmless but sometimes pathogenic. Thus, the liver tissue contains a broad collection of immune cells, including Kupffer cells (resident macrophages), T lymphocytes (LT) and natural killer (NK) cells, which represent 50% of total immune cells in the liver [12]. This hepatic innate and adaptive immune system is also involved in the recognition and destruction of cancerous cells [13]. However, during chronic liver disease, the constant established inflammation results in an increase of proinflammatory cytokine production (mostly the tumor necrosis factor (TNF-α) and the interferon gamma (IFN-γ)) and the recruitment of systemic immune cells. This unusual intrahepatic immune status promotes hepatocytes death, disease progression and contributes to carcinogenesis [14]. Once settled, tumors manage to control the anti-tumor functions of immune cells by producing immunosuppressive cytokines, such as the transforming growth factor beta (TGF-β) and IL-10 [15]. Besides, the intra-tumoral infiltration of immunosuppressive regulatory T cells (Tregs) is correlated with HCC progression and poor patient prognosis [16,17].

To better understand the impact of high calories diet composition and of the immune response on liver carcinogenesis and thus to allow the development of new therapeutics, relevant murine models are required. Fujii et al. have established a murine model enabling the study of HCC progression in a context of nonalcoholic steatosis. This model, based on diabetic C57Bl/6J mice fed with a high-fat diet (HFD), allowed systematic development of liver tumors after 16 weeks under this specific diet and is considered as a relevant model for HCC development studies [18].

In the present work, we investigated the physiopathological differences during liver cancer progression in diabetic mice fed with two different HFD. Diabetic mice fed either a diet similar to that used by Fujii et al., referred to herein as high-fat high-sugar diet (HFHSD) or on a high-fat high-cholesterol diet (HFHCD) were monitored for 16 weeks to evaluate steatosis, NASH severity and HCC development. The inflammatory status and immune response were also evaluated in both conditions during the course of the disease.

## 2. Results

### 2.1. HFD-Induced Steatosis Was Associated with Severe Liver Injuries Only for HFHCD

In order to compare NASH evolution according to the high calorie diet type, an experimental protocol using diabetic male mice was conducted. This protocol was described to induce chronic hepatitis leading to HCC development [18]. A type 1 diabetes mellitus was first triggered in C57Bl/6J male mouse pups using streptozotocin (STZ) treatment. Diabetic mice were then subjected, at the post-weaning stage and for 16 weeks, to HFD supplemented by an excess of cholesterol or of sugar (HFHCD or HFHSD, respectively). Two control groups of non-diabetic and diabetic mice were bred in parallel under a standard diet (SD). Body weight monitoring showed that, unlike the other groups, mice under HFHCD tended to lose weight at the time of diet-switch to remain stable thereafter (Figure S1a). This minor weight loss was probably due to, at least in part, the poor palatability of HFHCD, as revealed by diet consumption follow-up (Figure S1b). Indeed, calorie intake dropped to 10 kcal per day with HFHCD compared to a normal daily consumption of 15 kcal with the SD, a level maintained with HFHSD.

**Figure 1:**
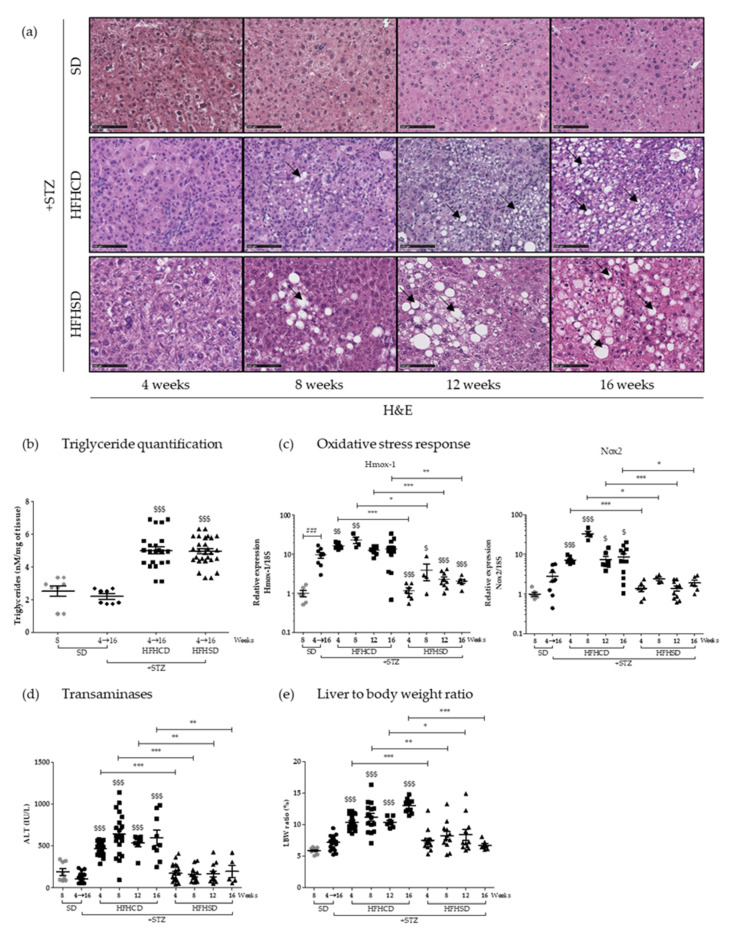
High-fat diet type influenced hepatitis severity in diabetic mice without affecting the steatosis grade. Healthy or diabetic (streptozotocin (STZ)) C57Bl6/J male mice were fed either a standard diet (SD), a high-fat-high-cholesterol diet (HFHCD) or a high-fat-high-sugar diet (HFHSD) during 4, 8, 12 or 16 weeks. (**a**) H&E staining of liver sections. Black arrows show lipid macrovesicles. Scale bars: 100 µm, original magnification ×200; (**b**) triglyceride concentrations measured in liver extracts; (**c**) hepatic mRNA expression levels of *Hmox-1* and *Nox2* genes; (**d**) plasma alanine transaminase (ALT) concentrations (IU/L) and (**e**) liver to body weight (LBW) ratio, percentage of liver weight on body weight. For all graph, each grey dots, black dots, black squares and black triangles represent individuals from the different groups: healthy mice under SD, diabetic mice under SD, diabetic mice under HFHCD or HFHSD, respectively. ### *p* < 0.001 compared diabetic mice under SD to healthy mice under SD; $ *p* < 0.05; $$ *p* < 0.01 and $$$ *p* < 0.001 compared diabetic mice under HFHCD or HFHSD to diabetic mice under SD; * *p* < 0.05; ** *p* < 0.01 and *** *p* < 0.001 compared diabetic mice under HFHCD to diabetic mice under HFHSD at the same time point.

The induced NAFLD starts with asymptomatic hepatic steatosis, a process followed on mice fed with HFHCD or HFHSD during 4, 8, 12 and 16 weeks on histological sections stained with hematoxylin-and-eosin (Figure 1a). Macrovesicular steatosis appeared as early as 8 weeks, and seemed to be larger in mice fed with HFHSD. However, triglyceride quantification did not show any differences between the two groups. When compared with control groups, the triglyceride levels doubled in the liver of mice under both HFD (Figure 1b).

During NAFLD evolution, oxidative stress sets and influences pathogenesis [19]. Two markers of the oxidative state of the hepatic tissue were analyzed by an RT-qPCR approach. Interestingly HFHCD-fed mice displayed significant higher levels of NADPH oxidase 2 (*Nox2*) and Heme oxygenase 1 (*Hmox-1*) mRNA than mice fed with HFHSD (Figure 1c). This difference was maintained throughout all the experimental kinetics, from the 4th to the 16th week. Worth noting that diabetes alone also caused some hepatic oxidative stress, a phenomenon already described [20]. To evaluate liver injury in parallel, serum transaminase levels were monitored over time. While alanine aminotransferase (ALT) concentrations remained consistently normal for mice under HFHSD, blood from HFHCD-fed animals showed higher concentrations from 4 weeks of diet, which persisted until the end of the protocol (Figure 1d). Such values (averaging at 500 and up to 1000 UI/L) revealed severe chronic liver damage, which were thus associated with an important oxidative stress, two typical NASH symptoms. Besides, only HFHCD also caused a sustained systematic hepatomegaly, as shown by the analysis of liver to the body weight ratio (Figure 1e).

These initial investigations showed that both tested hyper-caloric diets resulted in quantitatively identical steatosis in diabetic mice, but that the first explored symptoms of NASH appeared much more pronounced in the HFHCD-fed cohort.

### 2.2. The Fibrotic Stage Reached in the Livers of Diabetic Mice Differed According to the Type of HFD

**Figure 2:**
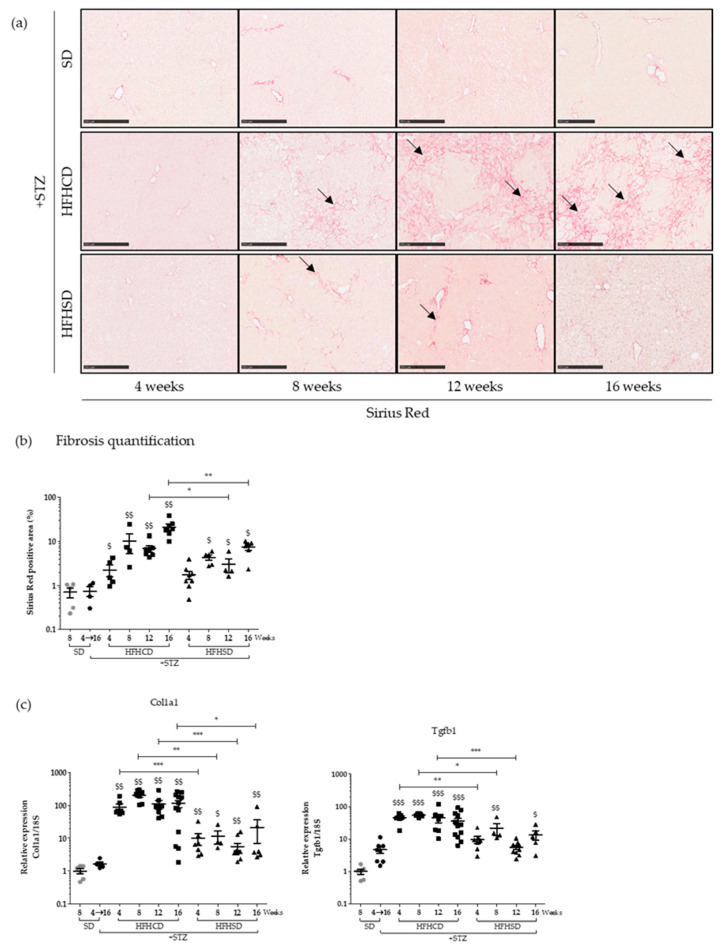
High-fat diet type affected fibrosis progress in diabetic mice. Healthy or diabetic (STZ) C57Bl6/J male mice were fed either a SD, a HFHCD or a HFHSD during 4, 8, 12 or 16 weeks. (**a**) Sirius Red staining of liver sections. Black arrows show collagen deposition. Scale bars: 250 µm, original magnification ×100; (**b**) quantification of fibrosis as determined by the percentage of Sirius Red positive area and (**c**) hepatic mRNA expression levels of *Col1a1* and *Tgfb1* genes. For all graph, each grey dots, black dots, black squares and black triangles represent individuals from the different groups: healthy mice under SD, diabetic mice under SD, diabetic mice under HFHCD or HFHSD, respectively. $ *p*<0.05; $$ *p* < 0.01 and $$$ *p* < 0.001 compared diabetic mice under HFHCD or HFHSD to diabetic mice under SD; * *p* < 0.05; ** *p* < 0.01 and *** *p* < 0.001 compared diabetic mice under HFHCD to diabetic mice under HFHSD at the same time point.

During NASH, the continuous presence of liver damage related to the chronic inflammatory state will activate stellate cells that will be responsible for the excessive deposition of the extracellular matrix, transforming the liver structure into scar tissue. Fibrosis was investigated in our models, directly within the tissue using Sirius Red staining (Figure 2a). Strikingly, large quantities of collagen fibers gradually accumulated in the liver of diabetic mice fed with HFHCD. In comparison, HFHSD only induced mild fibrosis. Signal quantification revealed that, in both cases, fibrosis began as early as week 4 and gradually intensified, with a much more pronounced slope for diabetic mice on HFHCD (Figure 2b). To support this histological data, the mRNA levels of genes known to be involved in fibrosis (collagen type I alpha 1 chain (*Col1a1*) and transforming growth factor beta 1 (*Tgfb1*)) were quantified in liver extracts. Both HFDs upregulated tested mRNA, with again more marked inductions in samples issued from HFHCD-fed diabetic mice (Figure 2c).

A new level of illness, fibrosis, had been rapidly reached in diabetic mice fed with HFDs. Thus, diabetic mice subjected to the tested HFDs developed a similar steatosis but a NASH syndrome of different severity. The evaluated physiopathological parameters (oxidative stress, liver injuries and fibrosis) remained mild for HFHSD but became severe for HFHCD.

### 2.3. Mild NASH, a Breeding Ground for HCC

**Figure 3:**
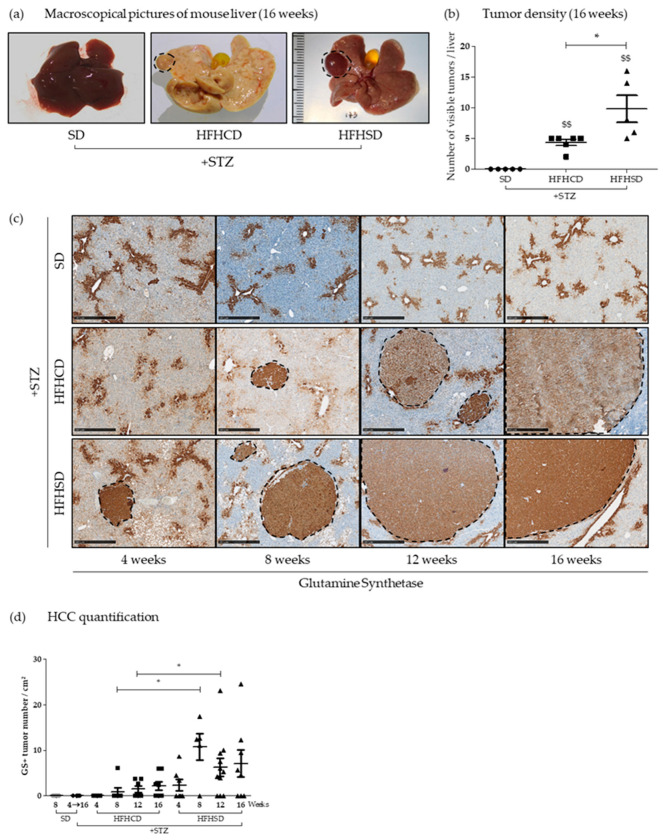
Nonalcoholic steatohepatitis (NASH)-driven hepatic carcinogenesis in diabetic mice depended on high-fat diet type. Healthy or diabetic (STZ) C57Bl6/J male mice were fed either a SD, a HFHCD or a HFHSD during 4, 8, 12 or 16 weeks. (**a**) Representative macroscopic liver pictures. Dashed lines delimit visible tumors; (**b**) tumor density measured from liver pictures and (**c**) glutamine synthetase staining of liver sections. Dashed lines delimit positive glutamine synthetase (GS+) tumors. Scale bars: 500 µm, original magnification ×50; (**d**) hepatocellular carcinoma quantification measured from GS+ area. For all graph, each grey dots, black dots, black squares and black triangles represent individuals from the different groups: healthy mice under SD, diabetic mice under SD, diabetic mice under HFHCD or HFHSD, respectively. $$ *p* < 0.01 compared diabetic mice under HFHCD or HFHSD to diabetic mice under SD; * *p* < 0.05 compared diabetic mice under HFHCD to diabetic mice under HFHSD at the same time point.

Livers of mice included in protocols were isolated and macroscopically analyzed. While the liver of control diabetic mice looked normal, those issued from diabetic mice fed for 16 weeks with one of the HFD displayed an abnormal morphology, with a granular surface, a stiffer texture and a pale color. This deterioration, typical of fibrotic livers, was much more pronounced in animals under HFHCD than under HFHSD (Figure 3a).

Macroscopic observation also revealed that some tumors protruded from the surface of the organ isolated exclusively from HFHSD- and HFHCD-fed diabetic mice. Their quantification showed more visible tumors on livers recovered from diabetic mice fed with HFHSD than those from mice fed with HFHCD (Figure 3b). Liver tissue sections were systematically stained with hematoxylin-and-eosin after 4, 8, 12 and 16 weeks of feeding with HFHCD or HFHSD for histological observation. Tumors, detected by changes in the liver histology, were identified in all diabetic mice under HFD after 8, 12 and 16 weeks of feeding (Figure S2a).

As glutamine synthetase (GS) has been described as an early biological marker of HCC [21], its immunostaining was performed on liver sections from all animal groups and at all times of kinetics (Figure 3c). Basal physiological staining of GS appeared in the centrilobular zone III on all tested specimen. As shown on Figure 3d, GS positive (GS+) nodules arose only in diabetic mice on HFD and never in SD-fed animals. The emergence of GS+ nodules began very early (at week 4) in diabetic mice fed with HFHSD, whereas the first GS+ nodules were detected only from week 8 in the liver of diabetic mice fed with HFHCD. In addition to the rapidity of their appearance in the liver of mice under HFHSD, the quantification of their density (number of GS+ nodules relative to the analyzed tissue surface) revealed that these livers contained a significantly higher number of GS+ nodules at 8 and 12 weeks of HFD. On the other hand, when comparing GS+ tumor sizes for same times of HFD, no difference emerged between the two experimental groups that received HFHCD or HFHSD (Figure S2b). These results indicated that in HFHCD mice, the liver environment seemed to limit tumor onset.

Despite the differences identified in carcinogenesis according to the type of HFD, the life expectancy of diabetic mice under HFHCD or HFHSD remained similar. Indeed, their respective survival curves showed no significant difference and was comparable to that already published [18] (Figure S2c). Besides, SD-fed diabetic mice had poorer survival, probably due to their high blood sugar level linked to the high carbohydrate intake (Figure S6a).

In conclusion, according to the HFD type, diabetic mice with mild hepatitis and fibrosis developed more tumors in their liver than diabetic mice suffering from severe hepatitis and fibrosis.

### 2.4. HFD-Composition Affected the Amplitude and Nature of the Immune Infiltrate

Distinct immunological liver microenvironments could explain the disparities observed between tumorigenesis occurring in diabetic mice fed with HFHCD or HFHSD. To initiate the analysis of the immune response elicited in the treated mice, liver sections stained with hematoxylin-and-eosin were studied for the presence of lobular inflammation and/or immune infiltrates (Figure S3). Lobular inflammation linked to steatohepatitis was only detected in diabetic mice fed with HFHCD and was associated to massive immune cell infiltrate from 8 to 16 weeks of feeding (Figure S3a). On the contrary, mild inflammation and only few spots of immune cells were observed on liver sections from diabetic mice fed with HFHSD. Hepatic inflammation was also revealed by an increase of tumor necrosis factor alpha (*Tnf-α*) and chemokine ligand 2 (*Ccl-2*) mRNA expression in diabetic mice fed with HFHCD only (Figure S3b).

**Figure 4:**
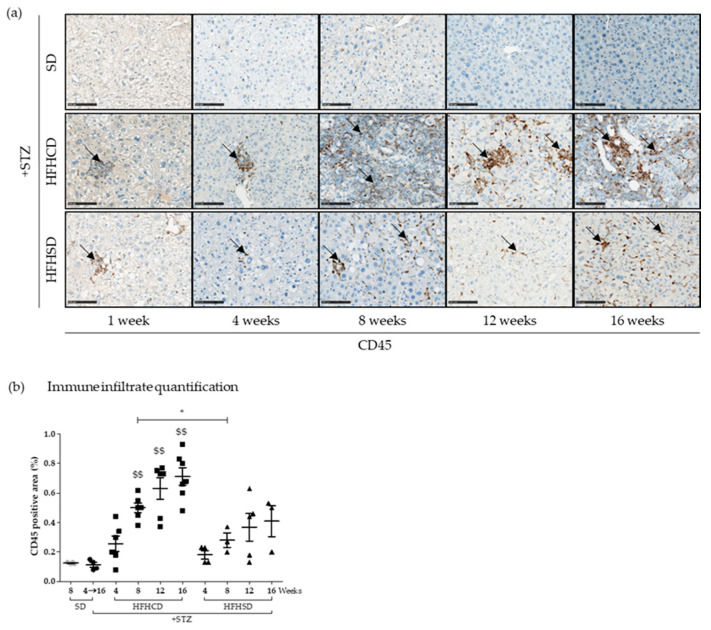
Immune infiltrate presence during hepatocellular carcinoma (HCC) development on NASH background. Healthy or diabetic (STZ) C57Bl6/J male mice were fed either a SD, a HFHCD or a HFHSD during 1, 4, 8, 12 or 16 weeks. (**a**) CD45 staining of liver sections. Black arrows show CD45+ immune infiltrates. Scale bars: 100 µm, original magnification ×100; (**b**) quantification of immune infiltrates measured from CD45 positive area. For the graph, each grey dots, black dots, black squares and black triangles represent individuals from the different groups: healthy mice under SD, diabetic mice under SD, diabetic mice under HFHCD or HFHSD, respectively. $$ *p* < 0.01 compared diabetic mice under HFHCD or HFHSD to diabetic mice under SD; * *p* < 0.05 compared diabetic mice under HFHCD to diabetic mice under HFHSD at the same time point.

In order to confirm immune cell presence in the livers of diabetic mice fed with both HFDs, immunochemistry staining was performed on tissue sections with an anti-CD45 antibody (Figure 4). CD45 is a surface marker shared by all leukocytes that is strongly expressed on lymphocytes and macrophages but weakly expressed on granulocytes. Immune cell infiltrates appeared in the livers of all diabetic mice fed with either HFHCD or HFHSD. However, tissues derived from HFHSD-fed diabetic mice exhibited less CD45 positive staining than those from HFHCD-fed mice (Figure 4a). Signal quantification showed that the level of immune cell infiltrates increased over time only significantly for diabetic mice fed with HFHCD when compared to tissues of control diabetic mice under SD (Figure 4b).

**Figure 5:**
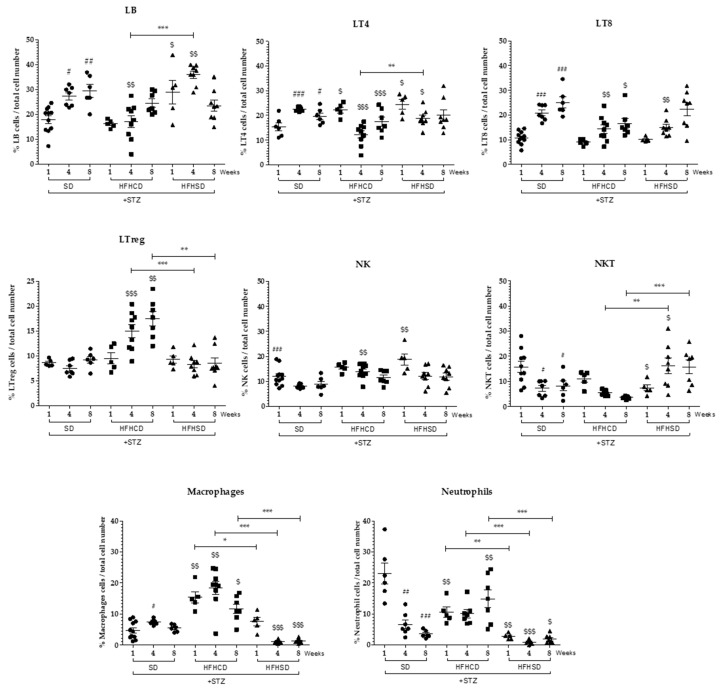
Analysis of hepatic immune infiltrates during HCC development on NASH background. Diabetic (STZ) C57Bl6/J male mice were fed either a SD, a HFHCD or a HFHSD during 1, 4 and 8 weeks. Percentages of B lymphocytes (LB), CD4+ T cells (LT4), CD8+ T cells (LT8), regulatory T cells (LTreg), Natural Killer (NK) cells, Natural Killer T (NKT) cells, macrophages and neutrophils in the different experimental conditions (percentage of total infiltrated immune cells). For all graph, each black dots, black squares and black triangles represent individuals from the different groups: diabetic mice under SD, diabetic mice under HFHCD or HFHSD, respectively. # *p* < 0.05; ## *p* < 0.01 and ### *p* < 0.001 compared diabetic mice under SD to healthy mice under SD; $ *p* < 0.05; $$ *p* < 0.01 and $$$ *p* < 0.001 compared diabetic mice under HFHCD or HFHSD to diabetic mice under SD; * *p* < 0.05; ** *p* < 0.01 and *** *p* < 0.001 compared diabetic mice under HFHCD to diabetic mice under HFHSD at the same time point.

To identify the cell infiltrate composition, hepatic immune cells were isolated from diabetic mouse livers after 1, 4 and 8 weeks of either HFHCD or HFHSD (Figure 5), characterized and quantified by flow cytometry (Figure S4a). Within live cells, B lymphocytes were defined as CD19+, T lymphocytes as CD19- CD3+ TCRαβ+ among which CD4+ Helper T cells, CD8+ Cytotoxic T cells and FoxP3+ Tregs were identified. Natural killer (NK) cells were defined as CD19-CD3-NK1.1+ when natural killer T (NKT) cells were CD-19-CD3+NK1.1+. Macrophages and granulocytes were identified within the non-lymphoid (CD19-CD3-NK1.1-) CD11b+ myeloid cells compartment and distinguished as respectively Gr1^intermediate^ or Gr1^high^. All these immune cell populations and their state of activation through the level of expression of CD69 were evaluated beforehand in the liver of healthy non-diabetic mice fed with SD (Figure S4b,c).

The diabetic status of mice alone led to a progressive increase in the number of CD8+ T cells, a situation found in all diabetic mice fed either with SD, HFHCD or HFHSD (Figure 5) as already described in previous published data [22]. In addition, a decrease in NKT cells and neutrophils was observed in diabetic mice fed with SD (Figure 5).

**Figure 6:**
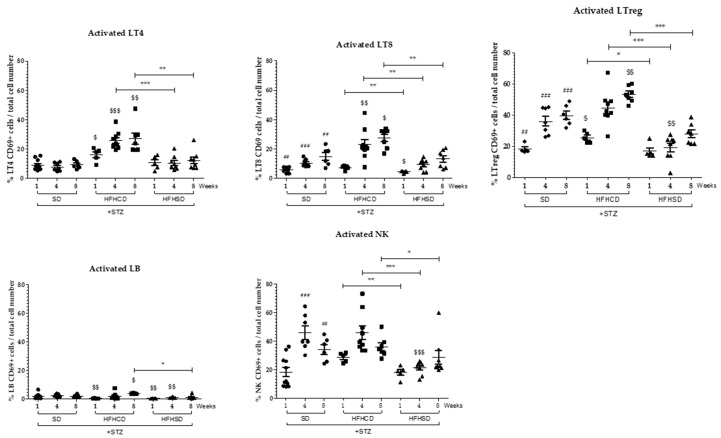
Analysis of the activation of hepatic immune infiltrates during HCC development on NASH background. Diabetic (STZ) C57Bl6/J male mice were fed either a SD, a HFHCD or a HFHSD during 1, 4 and 8 weeks. Percentages of activated CD4+ T cells (Activated LT4), CD8+ T cells (Activated LT8), regulatory T cells (Activated LTreg), B cells (Activated LB) and Natural Killer (Activated NK) cells. For all graph, each black dots, black squares and black triangles represent individuals from the different groups: diabetic mice under SD, diabetic mice under HFHCD or HFHSD, respectively. ## *p* < 0.01 and ### *p* < 0.001 compared diabetic mice under SD to healthy mice under SD; $ *p* < 0.05; $$ *p* < 0.01 and $$$ *p* < 0.001 compared diabetic mice under HFHCD or HFHSD to diabetic mice under SD; * *p* < 0.05; ** *p* < 0.01 and *** *p* < 0.001 compared diabetic mice under HFHCD to diabetic mice under HFHSD at same time point.

A significant recruitment of macrophages arose in the liver of HFHCD-fed diabetic mice. Concomitantly with this recruitment, CD4+ T cells and CD8+ T cells were only activated in the liver of mice fed with HFHCD from week 4 (Figure 5 and Figure 6). Regarding Tregs, which own immunosuppressive functions and thus control the immune response, their number and their activation state increased at week 4 and further at week 8 in the liver of diabetic mice fed with HFHCD (Figure 5 and Figure 6). These mice displayed increased activated NK cells when NKT cells gradually disappeared from livers, (as previously reported [23]), showing NASH progression in mice fed with HFHCD.

On the contrary, no drastic change occurred in the hepatic immune cell population of HFHSD-fed diabetic mice, either on their number or on their activation status.

Altogether, these data confirm the inflammatory status of the livers from HFHCD-fed mice and indicate that a specific immune response happen with activated cytotoxic T cells. The immune system was less solicited in the liver of mice under HFHSD since they showed no evidence of hepatic inflammation, or important recruitment of macrophages, or activation of T lymphocytes or a decrease in NKT cells.

## 3. Discussion

An inappropriate feeding behavior can lead to steatosis (hepatic fat accumulation), liver inflammation or even HCC. To evaluate the impact of HFD composition on the progression of these pathologies, two groups of diabetic mice were fed with two distinct HFDs, one supplemented with cholesterol and cholate (HFHCD) and the other enriched in sugar (HFHSD). The induction of diabetes in itself led to overconsumption in mice, from a daily intake of 3 g of SD to almost 5 g of SD. The low palatability of HFHCD was most likely responsible, at least in part, for the observed drop in daily food intake by diabetic mice, reaching only 1.6 g. Finally, while the calorie intake of lipid origin represented a consumption of 1.8 kcal per day for SD-fed diabetic mice, that of diabetic mice on HFHCD or HFHSD reached 9 and 9.2 kcal per day, respectively (Figure S1b). The indistinguishable steatosis levels measured in livers of diabetic mice fed with HFHCD or HFHSD may be explained by this similar high caloric intake of lipid origin.

HFHSD contained high percentages of carbohydrates, which, when combined with HFD, added a risk factor to develop HCC [18], and could contribute to tumors expansion in mice fed with HFHSD. Some studies showed that carbohydrates are associated with liver damage, NAFLD and HCC [24,25,26]. In liver, carbohydrates stimulate lipogenesis that generates high levels of adenosine triphosphate (ATP) and citrate, and induce fatty acids and triglycerides biosynthesis [27]. The high carbohydrate levels in HFHSD most likely contributed to steatosis hepatic damage, leading to hepatocyte proliferation, mild fibrosis and cancer development.

Not only did the proportions of lipid, carbohydrates and proteins differ between used diets but also the type of fatty acids (Figure S6). Thus, HFHCD contains more saturated fatty acid (SFA) than HFHSD, while there are more polyunsaturated fatty acids (PUFA) in HFHSD. SFA accumulation has been shown to induce hepatic toxicity and endoplasmic reticulum stress that can stimulate the proinflammatory profile of macrophages [28,29]. On the contrary, PUFAs are described to promote the prevention and the resolution of tissue inflammation [29]. Thus, the inflammatory profile of macrophages may depend on the ratio between SFA and PUFA. Accordingly, the SFA:PUFA ratio was much higher in HFHCD (17.35) than in HFHSD (2.31). This excess of SFA in HFHCD most probably contributed to the severe symptoms (inflammation and fibrosis) observed in diabetic mice under this diet [30]. Moreover, since SFA have also been described to participate in CD4+ T cell activation [29], their overabundance in HFHCD should contribute to the increase of CD69 expression on CD4+ T cells observed in the livers of mice fed with this diet.

Besides, cholate and cholesterol, which enrich HFHCD, would also take part in its detrimental impact since these compounds have been described to exacerbate inflammation and fibrosis [31,32,33]. Cholesterol is also, involved in cell death. This lipotoxic molecule increases the release of cytochrome c and of adenosine triphosphate (ATP) leading to c-Jun N-terminal kinases 1 (JNK1) activation and cell death by apoptosis or necrosis [28,34].

During NASH progression, the excess of lipids and fatty acids leads to liver lipotoxicity and hepatocyte injuries that initiate inflammation. Hepatocyte injury results in the release of damage-associated molecular patterns (DAMPs), which could activate Kupffer cells. In addition, patients with NASH have impaired intestinal permeability and a higher incidence of bacterial overgrowth leading to pathogen-association molecular patterns (PAMPs) release in blood [35]. PAMPs, commonly release by bacteria, viruses, parasites and fungi but also lipids contained in HFDs, could be detected by Kupffer cells and also induce their activation [36]. DAMPs- or PAMPs-activated Kupffer cells produce proinflammatory (TNF-α, IL-6 and IL-1β) and chemotactic factors (CCL-2 and CCL-5), fuelling the inflammation and inducing immune cell recruitment [37]. Liver of HFHCD-fed diabetic mice exhibited an inflammatory reaction signature, with a strong expression of TNF-α and CCL-2. Thus, our histological analyses confirmed the presence of numerous immune cells resulting from active infiltration of CD45+ cells and some of which was proliferating in the liver (Ki67+ cells; data not shown). These hepatic infiltrates contained many macrophages, probably responsible for the strong activation of the lymphocyte population, especially found in this group of HFHCD-fed diabetic mice. In parallel, it was found that, compared to diabetic mice under HFHSD, those included in the experimental protocol using HFHCD developed less liver tumor nodules. In combination with NK cells, T lymphocytes (CD4+ helper T and CD8+ cytotoxic T cells) are known to be key players of the tumors immune surveillance [38]. Thus, the presence of a greater number of active immune cells involved in tumor immunosurveillance in the liver of NASH-diabetic mice coincided with a limited emergence of hepatic tumors, despite an advanced fibrotic stage. This observation supports the hypothesis that a weak recruitment and activation of invasive immune cells in the liver of diabetic mice fed with HFHSD allowed cancerous hepatocytes to persist, proliferate and give rise to numerous tumor nodules. In these mice, the absence of liver inflammation does not result in macrophages and lymphocytes recruitment and activation. In fact, a weak activating signal of CD4+ T cells and CD8+ T cells could lead to lymphocytes apoptosis [39]. These data supports the lack of immune surveillance inside liver tissue in mice fed with HFHSD, a suitable environment for tumors onset. This last in vivo experimental condition may reproduce a part of patients with NAFLD without advanced fibrosis, but who still develop HCC [40]. This subpopulation represents around 35% of NAFLD-associated HCC. Interestingly, larger tumors are also found in this subclass of clinical cases [41].

The massive influx of immune cells into the liver of HFHCD-fed diabetic mice also involved Tregs. These specialized lymphocytes are immunosuppressive cells and help to contain the immune response to prevent any drift risk towards autoimmune reactions. Conversely, their inhibitory functions could impede the protective functions of anti-tumor immune response, promoting cancerous cell expansion [38]. This murine model of HCC development in a NASH background on diabetic mouse under HFHCD will afford the opportunity to test a therapeutic strategy targeting Tregs, an approach described as a promising cancer treatment [42,43].

## 4. Materials and Methods

### 4.1. Animal Model and Chemicals Treatment

All experimental protocols on animals were conducted in compliance with French laws and the institution’s guidelines for animal welfare (authors were authorized to conduct animal experimentation by “La direction des Services Vétérinaires” (license M Samson, #A3523840), the project was authorized by the “Comité Régional d’Ethique d’Expérimentation Animal” (CREAA), license given by the “Ministère de l’Education Nationale et de la Recherche”, #5656-2016061401105051). Figure S5a depicts the general experimental plan. Diabetes mellitus was induced by a single subcutaneous injection of 200 µg of STZ (Sigma-Aldrich, Lyon, France #S0130) in C57Bl/6J male mice two days after birth. Four groups were designed: a control group under standard diet (SD) without STZ treatment, a STZ-treated group under standard diet (STZ+SD), a STZ-treated group under HFHCD (STZ+HFHCD) and a STZ-treated group under HFHSD (STZ+HFHSD). At weaning, the diabetic status of mice was checked (Figure S5b). Diabetic mice were fed ad libitum with a SD (2016, Teklad Diet, Envigo, Gannat, France) or HFHCD (#9G21, LabDiet, St. Louis, MO, USA) or HFHSD (TD.06414, Teklad Custom Diet, Envigo, Gannat, France) during 1, 4, 8, 12 or 16 weeks. HDHCD is a HFD supplemented with cholesterol and contains high percentage of saturated fatty acid. HFHSD is a HFD enriched with carbohydrates (Figure S6). This last diet is similar to that used in the initial work describing this murine model of HCC on NASH background [18]. Animals were housed in individual cages and bred in specific pathogen-free conditions in conventional animal facility with a 12 h dark light cycle.

### 4.2. Biochemical Parameters

All animals were monitored daily and weighed once a week. Glycemia tests using glucometer (Freestyle Optium Neo, Abbott, Chicago, IL, USA) were performed after weaning and at slaughtering with submandibular blood collection using a lancet (#GR-3.5mm, Goldenrod Animal Lancet, Bioseb Lab).

Serum alanine aminotransferase (ALT) transaminase levels were measured according to the International Federation of Clinical Chemistry and Laboratory Medicine primary reference procedures using an Olympus AU2700 Autoanalyser (Olympus Optical).

### 4.3. Histological Analysis

Mouse liver was collected after slaughtering. Liver fragments were fixed in 4% paraformaldehyde and embedded in paraffin. Sections of 4 µm were used for hematoxylin-and-eosin (H&E), Sirius Red and immunohistochemistry stainings. For immunolocalization of glutamine synthetase (GS, Abcam, ab73593, 1/100), or of CD45 (BioLegend, #103107, 1/30), tissue sections were dried 1 h at 58 °C, followed by antigen retrieval and incubated with the corresponding primary antibody in a Ventana automated staining platform (Ventana Medical Systems, Illkirch-Graffenstaden, France). Revelation of primary antibody was carried out using horseradish peroxidase (HRP)-conjugated secondary antibody (Dako, Agilent Technologies, Les Ulis, France) and DAB substrate kit (Ventana, #760-124). Slides were then counterstained with hematoxylin.

All paraffin-embedded liver sections were scanned with a digital slide scanner (Nanozoomer 2.0-RS, Hamamatsu Photonics, Massy, France) and files were analyzed with the NDP viewer 2.5 software (Hamamatsu). Quantification of Sirius Red, CD45 or glutamine synthetase positive signals was performed with an image analysis software (NIS-Element AR analysis software, Nikon, Tokyo, Japan).

### 4.4. Hepatic Triglyceride Quantification Assay

Fragments of mouse liver, collected after slaughtering and preserved at −80 °C after rapid freezing in liquid nitrogen, were weighed and lysed by a 5% NP40 buffer (#FNN0021, Invitrogen, Thermo Fisher Scientific, Illkirch, France) using an Ultra-Turrax® homogenizer. Triglycerides were solubilized at 90 °C for 3 min and cleared by centrifugation at 15,000 rpm for 2 min. The Triglyceride Assay Kit (ab65336, Abcam, Amsterdam, Netherlands) was used for quantification, according to manufacturer’s instructions.

### 4.5. RNA Analysis

Total RNA was extracted from frozen liver fragments using the Nucleospin RNA kit (#740955, Macherey-Nagel) and an Ultra-Turrax® homogenizer according to manufacturer’s instructions. After RNA quantification using the NanoDrop (ND-1000 Spectrophotometer), cDNA was synthetized using the SuperScriptTM II Reverse Transcriptase (#18064022, Invitrogen). Real-time quantitative PCR was performed using the fluorescent SYBR Green dye (Power SYBR® Green PCR Master Mix, Applied Biosystems, Thermo Fisher Scientific, Illkirch, France) and the CFX384 TouchTM Real-Time PCR Detection System (Bio-Rad, Marnes La Coquette, France). The primer sequences used for qPCR are detailed in Supplementary Table S1. Each measurement was performed in triplicate. The relative gene expression was normalized against the 18S gene expression. Healthy control mouse group under SD, without STZ treatment, was used as a reference for mRNA expression (control mRNA level was arbitrarily set at 1).

### 4.6. Liver Immune Cell Analysis by Flow Cytometry

In order to remove blood, mouse livers were flushed by perfusion with PBS through inferior vena cava in situ before collecting the whole liver. Then, livers were crushed on a 70 µm cell strainer (Falcon #352350, Thermo Fisher Scientific, Illkirch, France). Parenchymal cells were removed by sedimentation for 1 h. Liver immune cells were isolated with density separation by centrifugation on RPMI media (RPMI 1640 Media, Gibco, Thermo Fisher Scientific, Illkirch, France) with 35% Percoll (Percoll density gradient media, GE Healthcare, Life Sciences, Thermo Fisher Scientific, Illkirch, France). For each liver, red blood cells were lysed by a treatment with ammonium chloride potassium (ACK) buffer (155 mM NH_4_Cl, 10 mM KHCO_3_ and 0.01 mM EDTA). Cell suspensions were labeled for 30 min with LIVE/DEAD fixable green stain (#L34959, Life technologies, Thermo Fisher Scientific, Illkirch, France) to exclude dead cells. Cells were also incubated with an anti-CD16/CD32 antibody (2.4G2, BD Pharmingen, Le Pont de Claix, France) to block non-specific binding before staining with fluorochrome-conjugated antibodies described in Supplementary Table S2. Fluorescence of stained cells was measured on LSRFortessa ×20™ flow cytometer (BD Biosciences, Le Pont de Claix, France) and data were analyzed using Flowlogic software (MACS Miltenyi Biotec). Dead cells and doublets were excluded on the basis of live/dead labeling and forward/side scatter, respectively. The immuno-phenotyping used was made on the appropriate size-FSC (forward side scatter)/structure-SSC (side scatter) gates and was as follows: B-lymphocytes: CD19+/CD3- cells; T-lymphocytes: CD3+/TCRVβ+/NK1.1-/CD19- on which CD4+ and CD8+ were distinguished and Tregs as FoxP3+ within the TCD4+ population; NKT cells: CD3+/NK1.1+/CD19-; NK cells: CD3-/NK1.1+-/CD19-; within the myeloid CD19-CD3-NK1.1-CD11b+ cell population, neutrophils were selected as GR1^high^ and macrophages as GR1^int^ (Figure S4a). Lymphoid activation was studied by analyzing the expression level of CD69. We calculated the percentage of each immune cell population, by considering the sum of events of all immune cell populations analyzed (T, NK, NKT, B cells and granulocytes and macrophages) as 100% of the total immune cells.

### 4.7. Statistical Analysis

Data were expressed as means ± SEM for all mice treated similarly. Mean differences between two experimental groups were assessed using the non-parametric Mann–Whitney U-test. All statistical analysis was achieved with the GraphPad Prism5 software. Calculated *p* values are integrated on histograms and graphs. Significance is shown as follows: # *p* < 0.05, ## *p* < 0.01 and ### *p* < 0.001 (comparison between diabetic mice under SD to healthy mice under SD groups); $ *p* < 0.05, $$ *p* < 0.01 and $$$ *p* < 0.001 (comparison between STZ+SD and STZ+HFHCD or STZ+HFHSD groups) and * *p* < 0.05, ** *p* < 0.01 and *** *p* < 0.001 (comparison between STZ+HFHCD and STZ+HFHSD groups).

## 5. Conclusions

In this study, we investigated the impact of HFD composition on HCC development. STZ-induced diabetic male mice were fed with HFHCD or HFHSD. Our results showed that the physiopathological conditions leading to the development of HCC was different in mice according to the diet. Mice fed with HFHCD developed severe liver injury, inflammation and fibrosis. This proinflammatory liver environment induced massive immune cell infiltrates, which seemed to be able to partly control tumor onset. On the contrary, diabetic mice fed with HFHSD presented moderate liver injury and mild fibrosis. This hepatic microenvironment with low inflammation and weak immune cell infiltrates was propitious to tumor development. Finally, these two in vivo mouse models of HCC progression in diabetic mice could be useful to better understand the heterogeneity of liver tumors and are therefore relevant supports for evaluating new therapeutic approaches, such as immune therapy.

## Supplementary Materials

The following are available online at https://www.mdpi.com/2072-6694/12/6/1491/s1, Figure S1: Mice monitoring, Figure S2: HCC development and mouse survival, Figure S3: Liver immune infiltrates in diabetic mice under distinct HFDs, Figure S4: Liver immune infiltrates in diabetic mice under distinct HFDs, Figure S5: Experimental design of in vivo experiments, Figure S6: Diet characteristics, Table S1: Sequences of primers used for qPCR, Table S2: Fluorochrome-conjugated antibodies used for flow cytometry.

## References

[1] Bray F., Ferlay J., Soerjomataram I., Siegel R.L., Torre L.A., Jemal A. (2018). Global cancer statistics 2018: GLOBOCAN estimates of incidence and mortality worldwide for 36 cancers in 185 countries. CA A Cancer J. Clin..

[2] Fujiwara N., Friedman S.L., Goossens N., Hoshida Y. (2018). Risk factors and prevention of hepatocellular carcinoma in the era of precision medicine. J. Hepatol..

[3] Wu Y., Ding Y., Tanaka Y., Zhang W. (2014). Risk Factors Contributing to Type 2 Diabetes and Recent Advances in the Treatment and Prevention. Int. J. Med. Sci..

[4] Bhupathiraju S.N., Hu F.B. (2016). Epidemiology of Obesity and Diabetes and Their Cardiovascular Complications. Circ. Res..

[5] Da Silva H.E., Arendt B.M., Noureldin S.A., Therapondos G., Guindi M., Allard J.P. (2014). A Cross-Sectional Study Assessing Dietary Intake and Physical Activity in Canadian Patients with Nonalcoholic Fatty Liver Disease vs Healthy Controls. J. Acad. Nutr. Diet..

[6] Wehmeyer M.H., Zyriax B.-C., Jagemann B., Roth E., Windler E., Wiesch J.S.Z., Lohse A.W., Kluwe J. (2016). Nonalcoholic fatty liver disease is associated with excessive calorie intake rather than a distinctive dietary pattern. Medicine.

[7] Desai A., Sandhu S., Lai J.-P., Sandhu D.S. (2019). Hepatocellular carcinoma in non-Cirrhotic liver: A comprehensive review. World J. Hepatol..

[8] Adams L.A., Ratziu V. (2015). Non-Alcoholic fatty liver—Perhaps not so benign. J. Hepatol..

[9] Ouyang X., Cirillo P., Sautin Y., McCall S.J., Bruchette J.L., Diehl A.M., Johnson R.J., Abdelmalek M.F. (2008). Fructose consumption as a risk factor for non-alcoholic fatty liver disease. J. Hepatol..

[10] Zelber-Sagi S., Nitzan-Kaluski D., Goldsmith R., Webb M., Blendis L., Halpern Z., Oren R. (2007). Long term nutritional intake and the risk for non-alcoholic fatty liver disease (NAFLD): A population based study. J. Hepatol..

[11] Musso G., Gambino R., De Michieli F., Cassader M., Rizzetto M., Durazzo M., Fagà E., Silli B., Pagano G. (2003). Dietary habits and their relations to insulin resistance and postprandial lipemia in nonalcoholic steatohepatitis. Hepatology.

[12] Racanelli V., Rehermann B. (2006). The liver as an immunological organ. Hepatology.

[13] Eggert T., Greten T.F. (2017). Tumor regulation of the tissue environment in the liver. Pharmacol. Ther..

[14] Schreiber R.D., Old L.J., Smyth M.J. (2011). Cancer Immunoediting: Integrating Immunity’s Roles in Cancer Suppression and Promotion. Science.

[15] Makarova-Rusher O.V., Medina-Echeverz J., Duffy A.G., Greten T.F. (2015). The yin and yang of evasion and immune activation in HCC. J. Hepatol..

[16] Sakaguchi S. (2004). Naturally arising CD4+ regulatory t cells for immunologic self-Tolerance and negative control of immune responses. Annu. Rev. Immunol..

[17] Kobayashi N., Hiraoka N., Yamagami W., Ojima H., Kanai Y., Kosuge T., Nakajima A., Hirohashi S. (2007). FOXP3+ Regulatory T Cells Affect the Development and Progression of Hepatocarcinogenesis. Clin. Cancer Res..

[18] Fujii M., Shibazaki Y., Wakamatsu K., Honda Y., Kawauchi Y., Suzuki K., Arumugam S., Watanabe K., Ichida T., Asakura H. (2013). A murine model for non-alcoholic steatohepatitis showing evidence of association between diabetes and hepatocellular carcinoma. Med. Mol. Morphol..

[19] Yang J., Fernández-Galilea M., Martínez-Fernández L., González-Muniesa P., Pérez-Chávez A., Martinez J.A., Moreno-Aliaga M.J. (2019). Oxidative Stress and Non-Alcoholic Fatty Liver Disease: Effects of Omega-3 Fatty Acid Supplementation. Nutrients.

[20] Mohamed J., Nafizah A.H.N., Zariyantey A.H., Budin S.B. (2016). Mechanisms of Diabetes-Induced Liver Damage. Sultan Qaboos Univ. Med. J..

[21] Long J., Wang H., Lang Z., Wang T., Long M., Wang B. (2010). Expression level of glutamine synthetase is increased in hepatocellular carcinoma and liver tissue with cirrhosis and chronic hepatitis B. Hepatol. Int..

[22] Zhou T., Hu Z., Yang S., Sun L., Yu Z., Wang G. (2018). Role of Adaptive and Innate Immunity in Type 2 Diabetes Mellitus. J. Diabetes Res..

[23] Vasseur P., Dion S., Filliol A., Genet V., Lucas-Clerc C., Jean-Philippe G., Silvain C., Lecron J.-C., Piquet-Pellorce C., Samson M. (2017). Endogenous IL-33 has no effect on the progression of fibrosis during experimental steatohepatitis. Oncotarget.

[24] Tessitore A., Mastroiaco V., Vetuschi A., Sferra R., Pompili S., Cicciarelli G., Barnabei R., Capece D., Zazzeroni F., Capalbo C. (2017). Development of hepatocellular cancer induced by long term low fat-high carbohydrate diet in a NAFLD/NASH mouse model. Oncotarget.

[25] Basaranoğlu M., Basaranoglu G., Bugianesi E. (2015). Carbohydrate intake and nonalcoholic fatty liver disease: Fructose as a weapon of mass destruction. HepatoBiliary Surg. Nutr..

[26] Garbow J.R., Doherty J.M., Schugar R.C., Travers S., Weber M.L., Wentz A.E., Ezenwajiaku N., Cotter D.G., Brunt E.M., Crawford P. (2011). Hepatic steatosis, inflammation, and ER stress in mice maintained long term on a very low-carbohydrate ketogenic diet. Am. J. Physiol. Liver Physiol..

[27] Cantley L.C. (2013). Cancer, metabolism, fructose, artificial sweeteners, and going cold turkey on sugar. BMC Biol..

[28] Leroux A., Ferrere G., Godie V., Cailleux F., Renoud M.-L., Gaudin F., Naveau S., Prévot S., Makhzami S., Perlemuter G. (2012). Toxic lipids stored by Kupffer cells correlates with their pro-inflammatory phenotype at an early stage of steatohepatitis. J. Hepatol..

[29] Hubler M.J., Kennedy A.J. (2015). Role of lipids in the metabolism and activation of immune cells. J. Nutr. Biochem..

[30] Charlton M.R., Krishnan A., Viker K., Sanderson S., Cazanave S., McConico A., Masuoko H., Gores G. (2011). Fast food diet mouse: Novel small animal model of NASH with ballooning, progressive fibrosis, and high physiological fidelity to the human condition. Am. J. Physiol. Liver Physiol..

[31] Ibrahim S.H., Hirsova P., Malhi H., Gores G.J. (2015). Animal Models of Nonalcoholic Steatohepatitis: Eat, Delete, and Inflame. Dig. Dis. Sci..

[32] Vergnes L., Phan J., Strauss M., Tafuri S., Reue K. (2003). Cholesterol and Cholate Components of an Atherogenic Diet Induce Distinct Stages of Hepatic Inflammatory Gene Expression. J. Biol. Chem..

[33] Liang J.Q., Teoh N., Xu L., Pok S., Li X., Chu E.S.H., Chiu J., Dong L., Arfianti A., Haigh W.G. (2018). Dietary cholesterol promotes steatohepatitis related hepatocellular carcinoma through dysregulated metabolism and calcium signaling. Nat. Commun..

[34] Kutlu O., Kaleli H.N., Özer E. (2018). Molecular Pathogenesis of Nonalcoholic Steatohepatitis- (NASH-) Related Hepatocellular Carcinoma. Can. J. Gastroenterol. Hepatol..

[35] Ganz M., Szabo G. (2013). Immune and inflammatory pathways in NASH. Hepatol. Int..

[36] Akira S., Uematsu S., Takeuchi O. (2006). Pathogen Recognition and Innate Immunity. Cell.

[37] Grunhut J., Wang W., Aykut B., Gakhal I., Torres-Hernandez A., Miller G. (2018). Macrophages in Nonalcoholic Steatohepatitis: Friend or Foe?. Eur. Med. J. Hepatol..

[38] Sachdeva M., Chawla Y.K., Arora S. (2015). Immunology of hepatocellular carcinoma. World J. Hepatol..

[39] Crispe I.N. (2014). Immune tolerance in liver disease. Hepatology.

[40] Wong C.R., Nguyen M.H., Lim J. (2016). Hepatocellular carcinoma in patients with non-alcoholic fatty liver disease. World J. Gastroenterol..

[41] Bengtsson B., Stål P., Wahlin S., Björkström N.K., Hagström H. (2019). Characteristics and outcome of hepatocellular carcinoma in patients with NAFLD without cirrhosis. Liver Int..

[42] Chaudhary B., Elkord E. (2016). Regulatory T Cells in the Tumor Microenvironment and Cancer Progression: Role and Therapeutic Targeting. Vaccines.

[43] Togashi Y., Shitara K., Nishikawa H. (2019). Regulatory T cells in cancer immunosuppression—Implications for anticancer therapy. Nat. Rev. Clin. Oncol..

