# Supplementary material for: Hepatocellular Carcinoma Emergence in Diabetic Mice with Non-Alcoholic Steatohepatitis Depends on Diet and Is Delayed in Liver Exhibiting an Active Immune Response
Source: Cancers (Basel). 2020 Jun 8;12(6):1491. doi: 10.3390/cancers12061491 (PMC7352283; doi:10.3390/cancers12061491)
Supplement: Supplementary file 1 [file cancers-12-01491-s001.pdf]

Supplementary Materials

Hepatocellular Carcinoma Emergence in Diabetic Mice with Non-Alcoholic Steatohepatitis Depends on Diet and is Delayed in Liver Exhibiting an Active Immune Response

Mélanie Simoes Eugénio, Muhammad Farooq, Sarah Dion, Christelle Devisme, Céline Raguenes-Nicol, Claire Piquet-Pellorce, Michel Samson, Marie-Thérèse Dimanche-Boitrel and Jacques Le Seyec

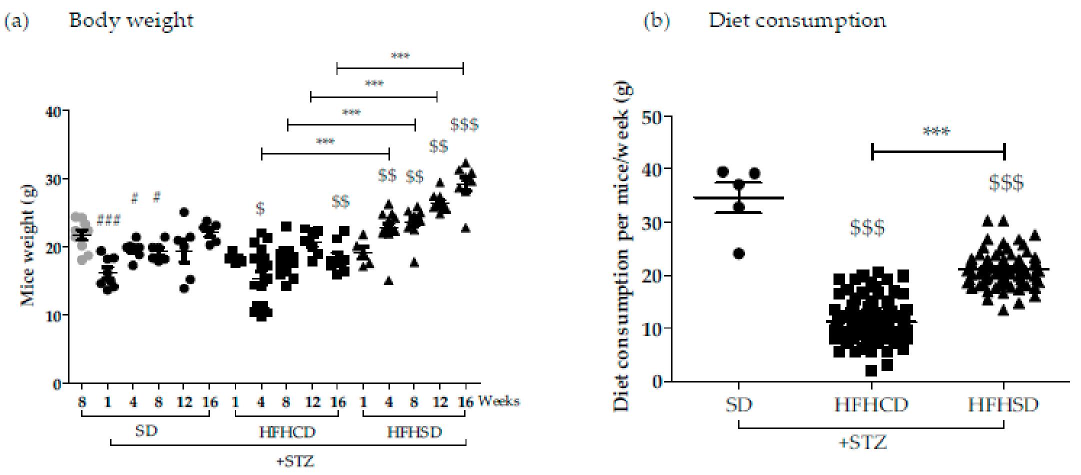

	SD	HFHCD	HFHSD
Daily food intake (g)	5	1.6	3
Daily calorie intake (Kcal)	15	10	15
Daily lipid calorie intake (Kcal)	1.8	9	9.2

Figure S1. Mice monitoring. Healthy or diabetic (STZ, Streptozotocin) C57Bl6/J male mice were fed either a standard diet (SD) or a high-fat diet (HFHCD, high-fat high-cholesterol diet; HFHSD, high-fat high-sugar diet) during 1, 4, 8, 12 or 16 weeks. (**a**) Mouse body weight; (**b**) Diet consumption per STZ treated mice per week (upper panel). Mean of daily food intake (g), daily calorie intake (kcal) and daily lipid calorie intake (kcal) for STZ-treated mice fed with SD, HFHCD or HFHSD. For all graph, each grey dots, black dots, black squares and black triangles represent individuals from the different groups: healthy mice under SD/diabetic mice under SD, diabetic mice under HFHCD or HFHSD, respectively. # $p < 0.05$; and ### $p < 0.001$ compared diabetic mice under SD to healthy mice under SD; $ $p < 0.05$; $$ $p < 0.01$ and $$$ $p < 0.001$ compared diabetic mice under HFHCD or HFHSD to diabetic mice under SD; *** $p < 0.001$ compared diabetic mice under HFHCD to diabetic mice under HFHSD at the same time point.

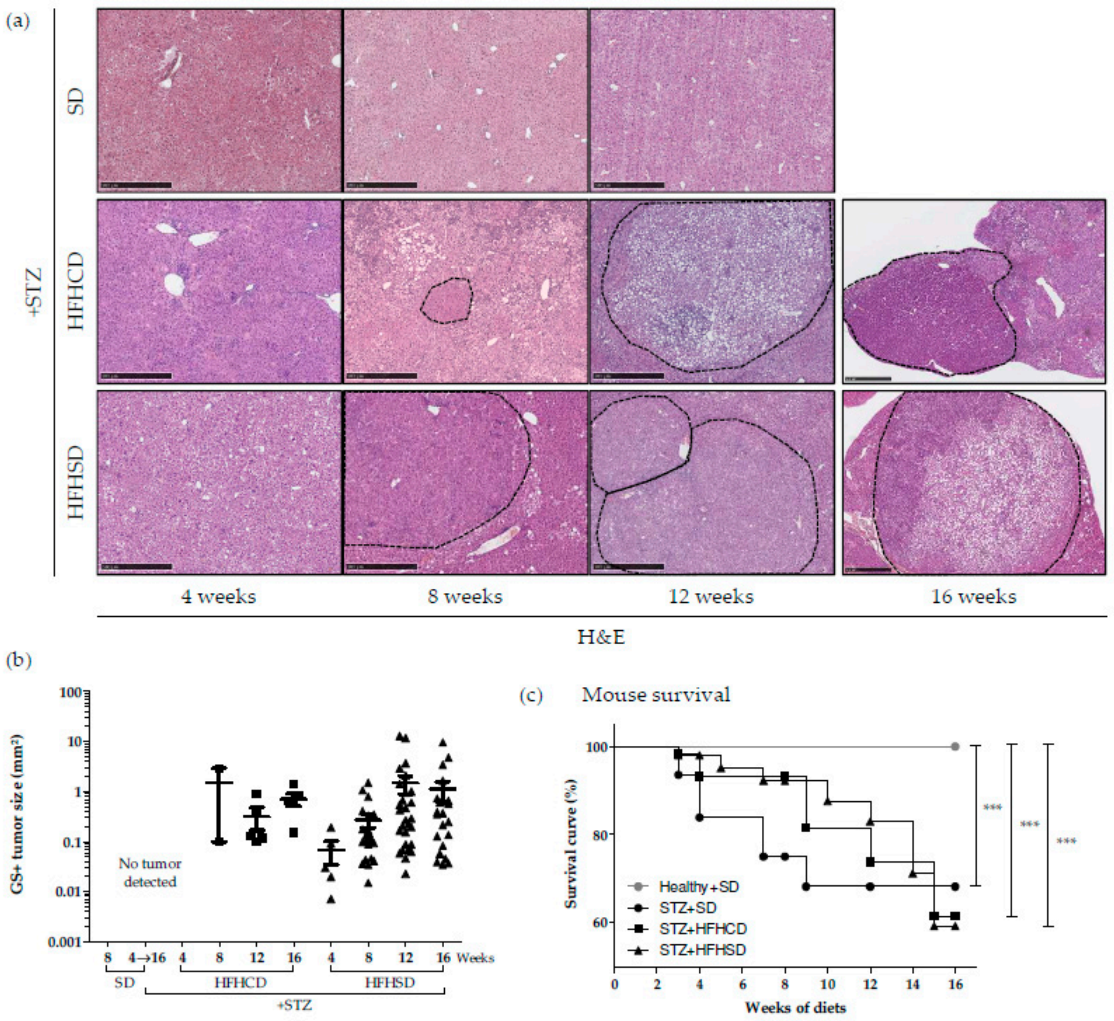

Figure S2. HCC development and mouse survival. Healthy or diabetic (STZ, Streptozotocin) C57Bl6/J male mice were fed either a standard diet (SD) or a high-fat diet (HFHCD, high-fat high-cholesterol diet; HFHSD, high-fat high-sugar diet) during 4, 8, 12 or 16 weeks. (**a**) Hematoxylin and-eosin (H&E) staining of liver sections. Dashed lines delimit tumor nodules. Scale bars: 500 µm, original magnification ×50; (**b**) Glutamine synthetase positive (GS+) tumor size (mm2), black squares and black triangles represent the different groups: healthy mice under SD, diabetic mice under SD, diabetic mice under HFHCD or HFHSD, respectively; (**c**) Mouse survival curves. *** $p < 0.001$ compared healthy mice under SD to diabetic mice under SD, HFHCD or HFHSD.

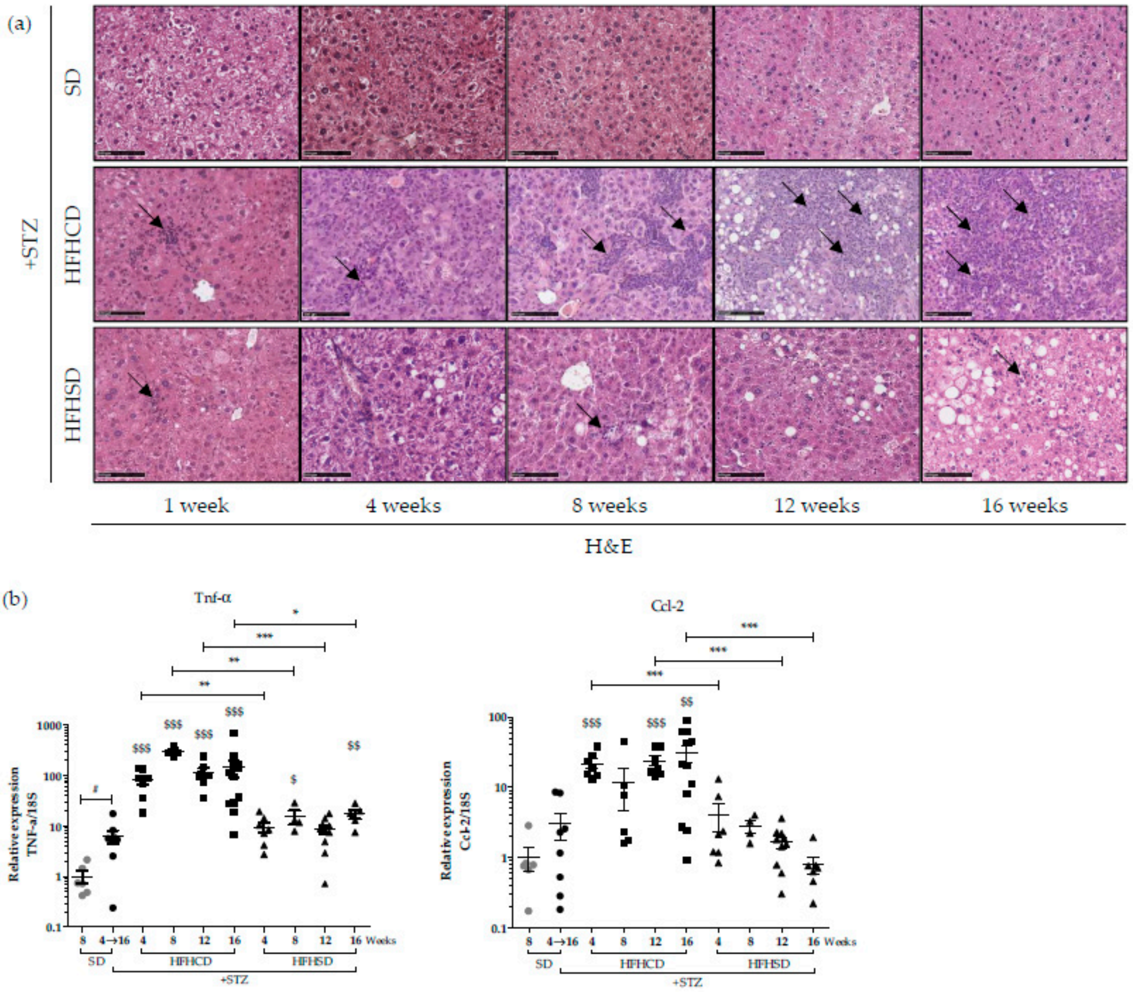

Figure S3. Liver immune infiltrates in diabetic mice under distinct HFDs. Healthy or diabetic (STZ, Streptozotocin) C57Bl6/J male mice were fed either a standard diet (SD) or a high-fat diet (HFHCD, high-fat high-cholesterol diet; HFHSD, high-fat high-sugar diet) during 1, 4, 8, 12 or 16 weeks. (**a**) Hematoxylin-and-eosin (H&E) staining of liver sections. Black arrows show visible immune infiltrates. Scale bars: 100 µm, original magnification x100; (**b**) Hepatic mRNA expression levels of Tnf-α and Ccl-2 genes. For all graph, each grey dots, black dots, black squares and black triangles represent individuals from the different groups: healthy mice under SD, diabetic mice under SD, diabetic mice under HFHCD or HFHSD, respectively. # $p < 0.05$ compared diabetic mice under SD to healthy mice under SD; \$ $p < 0.05$; \$\$ $p < 0.01$ and \$\$\$ $p < 0.001$ compared diabetic mice under HFHCD or HFHSD to diabetic mice under SD; * $p < 0.05$; ** $p < 0.01$ and *** $p < 0.001$ compared. Diabetic mice under HFHCD to diabetic mice under HFHSD at the same time point.

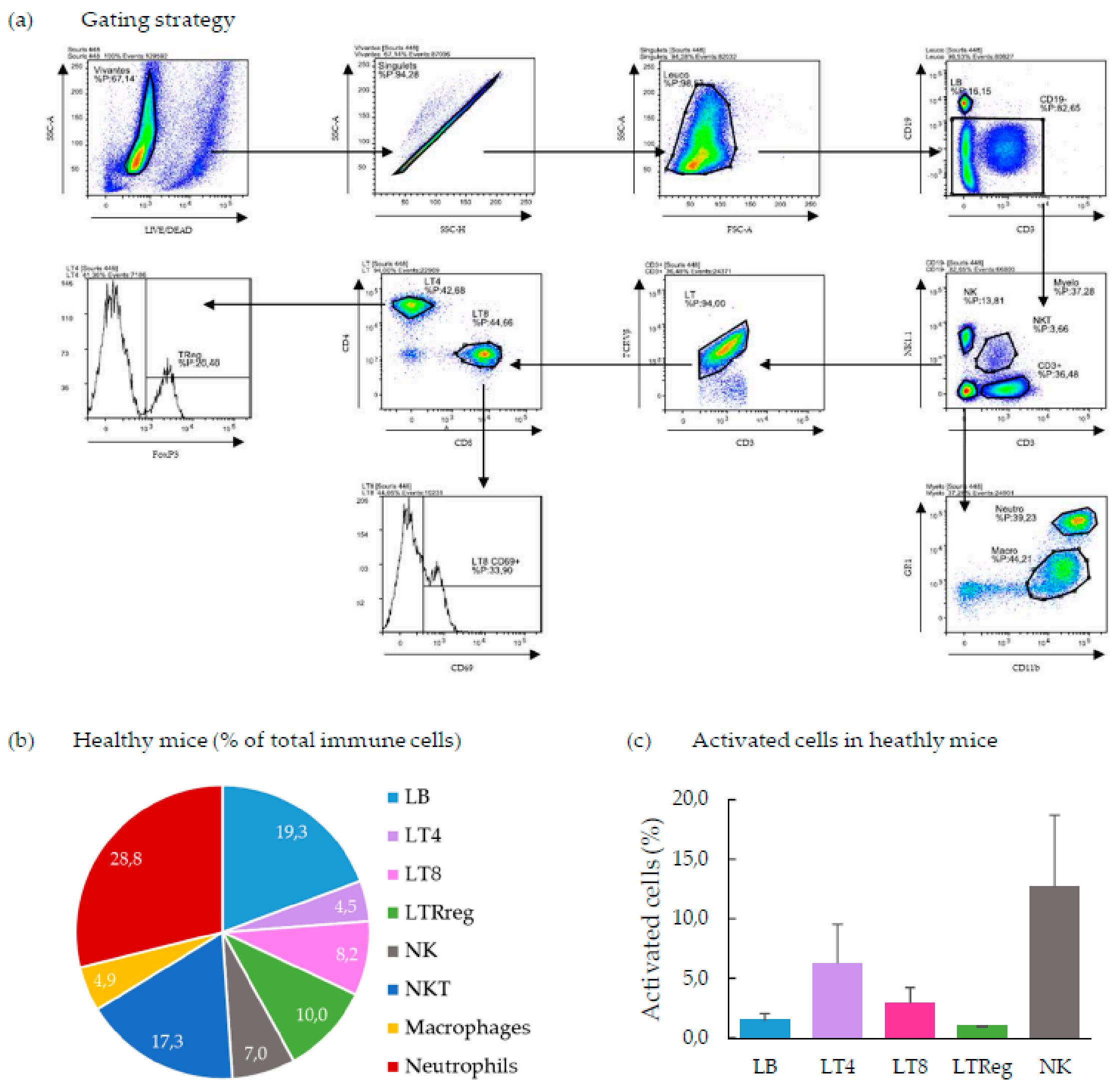

Figure S4. Liver immune infiltrates in diabetic mice under distinct HFDs. (**a**) Gating strategy used to identify the different populations of hepatic immune cells. Lymphocytes B (CD19+/CD3-), Lymphocytes T-CD8+ (CD3+/TCRVβ+/NK1.1-/CD8+), Lymphocytes T-CD4+ (CD3+/TCRVβ+/NK1.1-/CD4+), Regulatory T cells (CD3+/TCRVβ+/NK1.1-/CD4+/FoxP3+), NKT cells (CD3+/TCRVβ+/NK1.1+), NK cells (CD3-/NK1.1+), neutrophils (GR1+/CD11b+) and macrophages (GR1+/-/CD11b+) were first gated for singlets and on a viability marker. CD69+ marker were used to define LB, LT4, LT8 and NK activated cells; (**b**) Lymphocytes B (LB; light blue), Lymphocytes T-CD4+ (LT4; purple), Lymphocytes T-CD8+ (LT8; pink), Regulatory T cells (LTReg; green), Natural killer cells (NK; grey), Natural Killer T cells (NKT; dark blue), Macrophages (orange) and Neutrophils (red) proportion in liver of healthy mice under SD (% of total cells); (**c**) % of activated Lymphocytes B (LB; light blue), Lymphocytes T-CD4+ (LT4; purple), Lymphocytes T-CD8+ (LT8; pink), Regulatory T cells (LTReg; green), Natural killer cells. (NK; grey) in liver of healthy mice under SD.

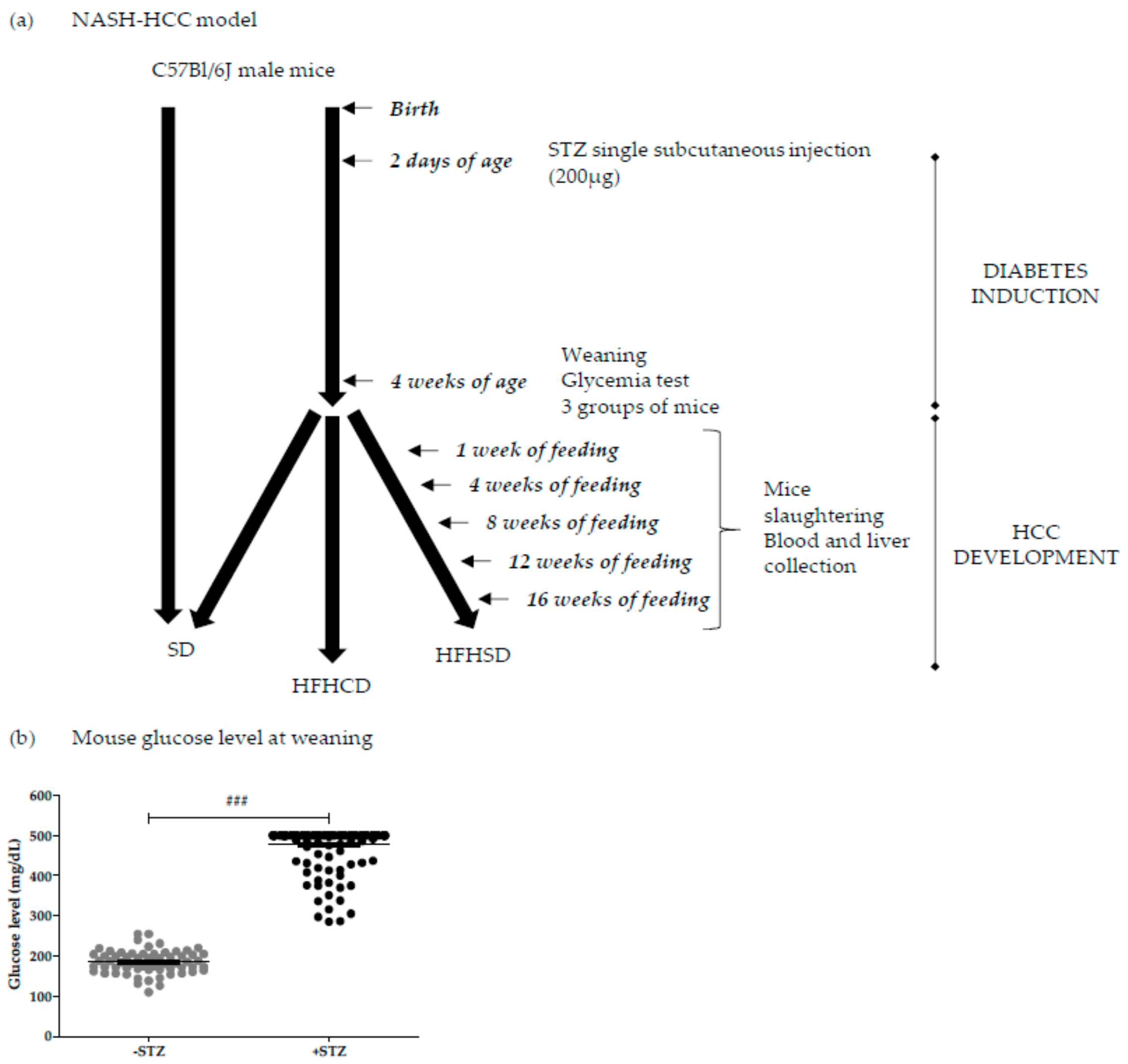

Figure S5. Experimental design of in vivo experiments. Two days old C57Bl/6J male mice were subcutaneously injected with streptozotocin (STZ). After weaning, diabetes status were validated and diabetic mice were fed either a standard diet (SD) or a high-fat diet (HFHCD, high-fat high-cholesterol diet; HFHSD, high-fat high-sugar diet) during 1, 4, 8, 12 or 16 weeks. (**a**) Experimental protocol; (**b**) Serum glucose levels of mice at weaning. For the graph, each grey and black dots represent individuals from the different groups: healthy (-STZ) and STZ-treated (+STZ) mice before feeding with SD, HFHCD or HFHSD diets. ### $p < 0.001$ compared STZ-treated mice under SD to healthy mice under SD.

(a) SD, HFHCD and HFHSD composition in proteins, fat and carbohydrates

	SD	HFHCD	HFHSD
Energy density (Kcal/g)	3	6,5	5,1
Protein	22%	6%	18%
Fat	12%	86%	60%
Carbohydrates	66%	8%	22%

(b) SD, HFHCD and HFHSD composition in fat

	SD	HFHCD	HFHSD
Total Fat (% by weight)	4	61,9	34,3
Total saturated fatty acid	0.6	36.4	12.7
Total monounsaturated fatty acid	0.7	20.4	16.1
Polyunsaturated fatty acid	2.1	2.1	5.5
Cholesterol	0	1,5	0

Figure S6. Suppliers provided diet compositions. (**a**) Energy density (Kcal/g) with energetic part of protein, carbohydrates and fat of standard diet (SD), high-fat high-cholesterol diet (HFHCD) and high-fat high-sugar diet (HFHSD); (**b**) Composition in lipids (% by weight) of standard diet (SD), high-fat high-cholesterol diet (HFHCD) and high-fat high-sugar diet (HFHSD).

Table S1. Sequences of primers used for qPCR.

Gene	Forward Primer (5′-3′)	Reverse Primer (5′-3)
18s	TTGGCAAATGCTTTCGCTC	CGCCGCTAGAGGTGAAATTC
Nox2	AGTGCGTGTTGCTCGACAA	GCGGTGTGCAGTGCTATCAT
Hmox1	AGGTACACATCCAAGCCGAGA	CATCACCAGCTTAAAGCCTTCT
Col1a1	GCTCCTGCTCCTCTTAGGG	GCAGAAAGCACAGCACTCG
Tgfb1	CACCATCCATGACATGAACC	CAGAAGTTGGCATGGTAGCC
Tnf-α	TAGCTCCCAGAAAAGCAAGC	TTTTCTGGAGGGAGATGTGG
Ccl-2	TCCCAATGAGTAGGCTGGAG	TCTGGACCCATTCCTTCTTG

Table S2. Fluorochrome-conjugated antibodies used for flow cytometry.

Antigen (Conjugate)	Clone	Reference
**GR1 (efluor 450)**	RB6-8C5	#48-5931-82, eBioscience
**CD4 (BV510)**	RM4-5	#561099, BD Pharmingen™
**TCR (BV605)**	H57-597	#562840, BD Pharmingen™
**CD19 (BV786)**	ID3	#563333, BD Pharmingen™
**CD69 (PercpCy5.5)**	H1.2F3	#104522, Biolegend
**FoxP3 (PE)**	150D/E4	#12-4774-42, eBioscience
**NK1.1 (PEefluor)**	PK136	#61-5941-80, eBioscience
**CD11b (PECy7)**	M1/70	#552850, BD Pharmingen™
**CD3 (APC)**	145.2.C11	#17-0031-81, eBioscience
**CD8 (APCCy7)**	53-6-7	#557654, BD Pharmingen™

© 2020 by the authors. Licensee MDPI, Basel, Switzerland. This article is an open access article distributed under the terms and conditions of the Creative Commons Attribution (CC BY) license (http://creativecommons.org/licenses/by/4.0/).
